# Immediate screening method for predicting the necessity of massive transfusions in trauma patients: a retrospective single-center study

**DOI:** 10.1186/s40560-014-0054-3

**Published:** 2014-09-10

**Authors:** Tetsuya Yumoto, Atsuyoshi Iida, Takahiro Hirayama, Kohei Tsukahara, Naoki Shiba, Hideo Yamanouchi, Keiji Sato, Toyomu Ugawa, Shingo Ichiba, Yoshihito Ujike

**Affiliations:** Advanced Emergency and Critical Care Medical Center, Okayama University Hospital, 2-5-1 Kita-ku, Shikata-cho, Okayama-shi, Okayama 700-8558 Japan

**Keywords:** Massive transfusion, Shock index, Base excess, Focused assessment of sonography for trauma

## Abstract

**Background:**

Hemostatic resuscitation might improve the survival of severely injured trauma patients. Our objective was to establish a simplified screening system for determining the necessity of massive transfusions (MT) at an early stage in trauma cases.

**Methods:**

We retrospectively analyzed the cases of trauma patients who had been transported to our institution between November 2011 and October 2013. Patients who were younger than 18 years of age or who were confirmed to have suffered a cardiac arrest at the scene or on arrival were excluded. MT were defined as transfusions involving the delivery of ≥10 units of red blood cell concentrate within the first 24 h after arrival.

**Results:**

A total of 259 trauma patients were included in this study (males: 178, 69%). Their mean age was 49 ± 20, and their median injury severity score was 14.4. Thirty-three (13%) of the patients required MT. The presence of a shock index of ≥1, a base excess of ≤ −3 mmol/L, or a positive focused assessment of sonography for trauma (FAST) result was found to exhibit sensitivity and specificity values of 0.97 and 0.81, respectively, for predicting the necessity of MT. Furthermore, this method displayed an area under the receiver operating characteristic curve of 0.934 (95% confidence interval, 0.891–0.978), which indicated that it was highly accurate.

**Conclusions:**

Our screening method based on the shock index, base excess, and FAST result is a simple and useful way of predicting the necessity of MT early after trauma.

## Background

Uncontrolled hemorrhaging is a major cause of death in trauma patients [1]. In addition to undergoing surgical intervention or angiographic embolization to control the bleeding, patients who suffer such hemorrhaging can also require massive transfusions (MT). Damage control resuscitation (DCR), which integrates permissive hypotension, hemostatic resuscitation, and damage control surgery is a crucial strategy for treating severely injured trauma patients [2,3]. Damage control-based surgery focuses on controlling bleeding and contamination, and hemostatic resuscitation aims to manage coagulopathy as soon as possible via the early induction of MT protocols involving a balanced ratio of blood products and restrictive fluid replacement to prevent the development of coagulopathy [4,5]. Although several models for predicting the necessity of MT have been reported [6-9], they are too complicated for practical use. The aim of this study is to establish a simple screening method for predicting the necessity of MT at a very early stage in trauma cases.

## Methods

### Study population

Data for traumatically injured patients who were transported to Okayama University Hospital between November 1, 2011 and October 31, 2013 were retrospectively collected. Patients who were younger than 18 years of age or were confirmed to have suffered a cardiac arrest at the scene or on arrival at the emergency department (ED) were excluded. This study was approved by the institutional review board at the Okayama University.

### Definition of massive transfusions

MT were defined as transfusions of ≥10 units of red blood cell concentrate (RCC) that were administered within the first 24 h after the patient’s arrival at hospital. Patients who were bleeding and were expected to require high-volume transfusions within 24 h, but did not survive for 24 h, were also defined as having required MT in order to reduce survivor bias [7,10]. After the source of bleeding had been identified and hemorrhaging had been controlled via surgical or catheter intervention, the necessity of MT was determined based on clinical judgments. The patients who required MT received transfusions of RCC, fresh frozen plasma (FFP), and platelet concentrates (PC) at a 1:1:1 ratio. RCC, FFP, and PC were administered to maintain a hemoglobin level of ≥7.0 g/dl, an international normalized ratio (INR) of ≤1.5, a fibrinogen level of ≥200 mg/dl, and a platelet count of ≥5 × 10^4^/μl on repeated laboratory examinations.

### Data collection

The following data were recorded: age; sex; the mechanism of injury (blunt or penetrating); heart rate; systolic blood pressure; shock index (SI, defined as the ratio of heart rate to systolic blood pressure); base excess (BE); serum lactate level; hemoglobin level on arrival; the results of focused assessments of sonography for trauma (FAST); the presence or absence of pericardial effusion, intrathoracic fluid, or intraabdominal fluid; the injury severity score (ISS); the total amount of transfused products delivered within 24 h; and the outcome at hospital discharge.

### Statistical analysis

Categorical variables are shown as frequencies or percentages, whereas continuous variables are presented as mean and standard deviation (SD) values or median and interquartile range values depending on their distributions. Categorical variables were compared using Fisher’s exact probability test. Student’s *t* test was used to assess continuous variables with normal distributions, and the Mann-Whitney *U*-test was used to evaluate variables with non-normal distributions. We used multiple logistic regression analysis to identify independent predictors of the necessity of MT. The ability of the resultant model to predict the necessity of MT was estimated based on the area under the receiver operating characteristic curve (AUROC). *P* values of <0.05 were considered to be statistically significant. All analyses were performed using the software SPSS for Windows (release 19.0).

## Results

A total of 259 trauma patients were included in this study (males: 178, 69%). Their mean age was 49 ± 20, and their median ISS was 14.4. Thirty-three (13%) patients required MT, and the overall mortality rate was 4.6% (*n* = 12). Of the 33 patients who required MT, two died within 24 h and did not actually receive MT (one due to hemorrhagic shock and the other one due to a severe traumatic brain injury complicated with hemorrhagic shock). The causes of death in the seven patients who died after receiving MT were as follows: catastrophic brain injury (two patients), severe traumatic brain injury complicated with hemorrhagic shock (four patients), and exsanguination (one patient). The baseline characteristics of the MT and non-MT groups are shown in Table 1. In the MT group, the median total amounts of RCC, FFP, and PC administered were 20, 12, and 20 units, respectively. Among these predictors of the necessity of MT, we subjected a high shock index, a reduced BE, lower level of hemoglobin, and a positive FAST result to multivariate analysis, as clinically important, these parameters can be assessed within a few minutes of arrival at the ED and so might be useful for establishing an easy and simplified screening method for determining the necessity of MT. Multivariate analysis revealed that a high shock index, a reduced BE, and a positive FAST result were predictors of MT being required (Table 2). Thus, we created a new simplified screening model based on these three predictors, i.e., a shock index of ≥1, a BE of ≤ −3 mmol/l, and a positive FAST result. In our scoring system, one point was awarded for each of these components, and hence, the total score ranged from 0 to 3. Shock index and BE cut-off points were determined based on AUROC values, as shown in Table 3.

**Table 1:** **Baseline characteristics of the MT and non-MT groups**

	**MT group**	**non-MT group**	***P*** **value**
Age (year)	53 ± 20	49 ± 20	0.22
Males, *n* (%)	24 (73)	154 (68)	0.60
Blunt mechanism, *n* (%)	30 (91)	228 (95)	0.25
HR (beats/min), mean ± SD	106 ± 31	87 ± 16	<0.001
SBP (mmHg), mean ± SD	99 ± 36	136 ± 27	<0.001
Shock index, median (IQR)	1.04 (0.79, 1.50)	0.63 (0.52, 0.77)	<0.001
Body surface temperature (°C), median (IQR)	36.1(35.7, 36.6)	36.6(36.1, 36.9)	0.002
BE (mmol/l), median (IQR)	−4.8 (−8.2, −2.4)	−0.3 (−2.2, 1.0)	<0.001
Lactate (mmol/l), median (IQR)	3.7 (2.8, 5.7)	2.0 (1.3, 2.7)	<0.001
Hemoglobin (g/dl), median (IQR)	12.4 (10.7, 13.4)	13.8 (12.3, 15.0)	<0.001
Platelet count (×104/μl), median (IQR)	19.3 (15.6, 22.3)	21.5 (18.1, 25.4)	0.013
Fibrinogen (mg/dl), median (IQR)	247 (192, 312)	324 (272, 376)	<0.001
INR, median (IQR)	1.02 (0.95, 1.37)	0.92 (0.88, 0.98)	<0.001
D-dimer (μg/ml), median (IQR)	32.0 (12.6, 101.8)	4.6 (0.9, 19.1)	<0.001
Positive FAST result, *n* (%)	20 (61)	16 (7)	<0.001
Pelvic fracture, *n* (%)	14 (42)	14 (6)	<0.001
ISS, median (IQR)	41 (30, 45)	9 (2, 17)	<0.001
RCC (units), median (IQR)	20 (13, 31)	0 (0, 0)	<0.001
FFP (units), median (IQR)	12 (8, 22)	0 (0, 0)	<0.001
PC (units), median (IQR)	20 (0, 45)	0 (0, 0)	<0.001
Mortality, *n* (%)	9 (27)	3 (1)	<0.001

Shock index: heart rate/systolic blood pressure. *MT* massive transfusion, *HR* heart rate, *SD* standard deviation, *SBP* systolic blood pressure, *IQR* interquartile range, *BE* base excess, *INR* international normalized ratio, *FAST* focused assessment of sonography for trauma, *ISS* injury severity score, *RCC* red cell concentrate, *FFP* fresh frozen plasma, *PC* platelet concentrate.

**Table 2:** **Multivariate analysis of predictors of massive transfusion**

**Variables**	**Odds ratio (95% CI)**	***P*** **value**
Shock index	259.81 (17.85–3782.41)	<0.001
BE	0.72 (0.58–0.89)	0.002
Hemoglobin	0.96 (0.76–1.22)	0.743
Positive FAST result	7.28 (2.24–23.61)	0.001

*CI* confidence interval, *BE* base excess, *FAST* focused assessment of sonography for trauma.

**Table 3:** **AUROC values obtained for our new screening method using different shock index and BE cut-off points**

**Shock index**	**BE**	**FAST**	**AUROC**	**95% CI**
≧1	≦ − 2.0	positive	0.919	0.869–0.968
≧1	≦ − 3.0	positive	0.934	0.891–0.978
≧1	≦ − 4.0	positive	0.928	0.875–0.981
≧0.8	≦ − 3.0	positive	0.909	0.858–0.960
≧1.5	≦ − 3.0	positive	0.895	0.831–0.959

*AUROC* area under the receiver operating characteristic curve, *BE* base excess, *FAST* focused assessment of sonography for trauma, *CI* confidence interval.

## Discussion

**Figure 1:**
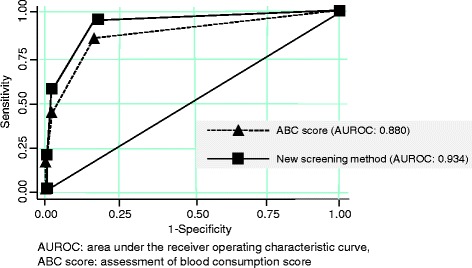
**AUROC values for the ABC scoring system and our new screening method.**

Exsanguination is the most frequent acute cause of death (51%) in trauma patients [1], and persistent hemorrhagic shock that requires MT eventually results in multiple organ failure [11]. DCR is an integrated approach used to treat severely injured trauma patients. In this method, damage control surgery, which is a surgical technique that focuses on controlling hemorrhaging and contamination, is first performed. Then, blood pressure is maintained below approximately 90 mmHg to prevent further bleeding, and multiple blood products and limited crystalloid fluid resuscitation are employed to treat and prevent coagulopathy. DCR must be initiated immediately after arrival in the ED and is continued in both the operating room and the intensive care unit [3,10,12,13]. As for MT, several studies have proposed predictive models for identifying trauma patients who require MT [6-9]. These models are each composed of several variables, e.g., basic data such as age and the mechanism of injury; vital signs such as heart rate and systolic blood pressure; laboratory data such as BE, serum lactate, hemoglobin, and INR values; and diagnostic imaging findings such as FAST results and parameters derived from X-rays of the pelvis. Among these models, the assessment of blood consumption (ABC) score, which is based on the mechanism of injury, heart rate, systolic blood pressure, and FAST results, was developed as a simplified model for predicting the necessity of MT early [7]. This score of ≥2 exhibits a sensitivity value of 75%, a specificity value of 86%, and a positive predictive value of 84%. However, when the ABC scoring system was applied to our data, an ABC score of ≥2 only exhibited a sensitivity value of 48% (Table 4). It is considered that the ABC score exhibits low sensitivity in Japan because blunt mechanisms account for the majority of injuries experienced in this country. Secondary, there were a total of 17 false negative patients using ABC score of ≥2. Of them, eight patients were complicated with severe traumatic brain injury. These patients have not necessarily presented with hypotension and tachycardia on arrival. These factors are associated with low sensitivity value of ABC score. As for our new screening model, 97% of patients who required MT met at least one of the abovementioned three conditions (Table 5). Figure 1 shows the AUROC of the ABC score and our new screening model. Our model exhibited a strong ability to predict the necessity of MT. In the presence of at least one of the three conditions, our model displayed high sensitivity and specificity for predicting the necessity of MT; however, its positive predictive value was only 43% (Table 5). Our screening method, which is based on the presence of a high shock index, a low BE value, and/or a positive FAST result, displayed high levels of accuracy during ROC analysis. In addition, this screening method can be completed within minutes of the patient’s arrival in the ED, which would allow MT protocols to be initiated quickly. Although our method exhibits high sensitivity and specificity, its positive predictive value is very low; thus, it might not be useful for diagnostic purposes.

**Table 4:** **The ABC scoring system and our new screening method**

	**Points**
ABC score	
Penetrating mechanism	1
SBP of ≤90 mmHg	1
HR of ≥120 bpm	1
Positive FAST result	1
Our new screening method	
Shock index of ≥1	1
BE of ≤ −3.0 mmol/L	1
Positive FAST result	1

*ABC score* assessment of blood consumption score, *FAST* focused assessment of sonography for trauma, *SBP* systolic blood pressure.

**Table 5:** **Sensitivity and specificity values and the percentage of correctly classified cases obtained with the ABC scoring system and our new screening method (Table** 4 **)**

	**Cut-off point**	**Sensitivity (%)**	**Specificity (%)**	**Correctly classified (%)**
ABC score in original study	≧0	100	0	13
	≧1	95	56	61
	≧2	75	86	84
	≧3	25	97	87
	≧4	6	100	88
ABC scores obtained using our center’s data	≧0	100	0	13
	≧1	85	85	46
	≧2	48	98	80
	≧3	19	100	100
	≧4	0	100	–
Our new screening method	≧0	100	0	13
	≧1	97	81	43
	≧2	61	93	56
	≧3	36	98	69

*ABC score* assessment of blood consumption score, *BE* base excess, *FAST* focused assessment of sonography for trauma, *SBP* systolic blood pressure, *HR* heart rate.

This was a single-center retrospective study involving a small population, and several other important limitations also exist. For example, our results might not be applicable to institutions that use different MT protocols or trauma systems or to different patient populations. Although our patients were managed with the aim of attaining a high FFP to RCC transfusion ratio, the actual ratio achieved was relatively low (0.6:1), which suggests that some patients might not have actually required MT. Several studies have reported that a high FFP to RCC ratio improves the outcomes of severely injured trauma patients, but we have to take survivor bias into consideration [14,15]. Another point is that some patients could have avoided MT if earlier or alternative methods have been employed to control their bleeding. Further investigation is necessary to determine whether our screening system facilitates the early initiation of MT protocols and the rapid control of hemorrhaging.

## Conclusions

Screening based on a combination of a high shock index, a low BE value, and/or a positive FAST result is an easy and useful way of predicting the necessity of MT in trauma patients; however, this approach might not be useful for diagnostic purposes.

## Abbreviations

MT: Massive transfusion
FAST: positive focused assessment of sonography for trauma
DCR: damage control resuscitation
ED: emergency department
RCC: red cell concentrations
FFP: fresh frozen plasma
PC: platelet concentrates
INR: international normalized ratio
SI: shock index
BE: base excess
ISS: injury Severity Score
AUROC: area under the receiver operating characteristic curve
SD: standard deviations
IQR: interquartile ranges
ABC: assessment of blood consumption
